# Lawrence M Field’s article on “An evolution of the ‘Golden Age’ of dermatological surgery (1958-2008)” and my views

**DOI:** 10.4103/0974-2077.44171

**Published:** 2008

**Authors:** Suresh P Joshipura

**Affiliations:** *Consultant Dermatologist and Medical Director of Research, H. J. Doshi Medical Research Foundation, Coins Corner, Dr. Yagnik Road, Rajkot 360 001, Gujarat, India. E-mail: suresh1ad1@gmail.com*

Sir,

I read with interest the above-mentioned article. Dermatology as a specialty has changed radically over the years. As a senior dermatologist who has watched these changes from close quarters, I wish to record my views:

There were days when dermatology used to be the last choice for a postgraduate course. The trend has completely changed these days. More and more graduates voluntarily opt for dermatology. Recently, it has been observed that a topper from AIIMS has chosen dermatology. This shows that dermatology is becoming a lead branch.

More and more lady dermatologists are entering our field, which was hitherto dominated by men. Formerly ‘obstetrics and gynecology’ was preferred by lady doctors. But because of lack of emergencies, fixed office hours, and lucrative income, dermatology is becoming the preferred branch for female doctors.

Cosmetology is a lucrative offshoot, not only of dermatology but also of plastic and general surgery, and sadly of the beauty shops as well; and sometimes such practice by those who are not qualified can lead to disastrous consequences.

Aesthetic and cutaneous surgery can be best undertaken by an experienced and trained dermatologist because only he/she knows the etiopathology, clinical diagnosis, and outcome of the therapy.

In India, dermatosurgery has had a long history. Veteran dermatologist Lt. Dr. P. N. Behl used to teach vitiligo surgery since the 1960s and was one among the initial dermatologists to perform this surgery. After the foundation of the Association of Cutaneous Surgeons of India (ACSI) in 1995, many workshops were organized; and gradually, inclination to learn, and awareness about cutaneous surgery increased among dermatologists. Prior to formation of ASCI, there were workshops on cutaneous surgery organized at the National Conference of the Indian Association of Dermatologists, Venereologists, Leprologists (DERMACONS). Advancement in instrumentation and procedures is rapid, making it often difficult to keep pace.

However, this maddening pace should not prevent us from recognizing the importance of clinical dermatology, which is the basis of procedures for esthetic and cutaneous surgery. While teaching postgraduate students, it is imperative that teachers of dermatology strongly emphasize the need to learn scientific, basic, clinical dermatology before embarking on cosmetic cutaneous surgery.

The author strongly feels that first, clinical dermatology should be learnt thoroughly – totally independent of cosmetology. Only after obtaining a degree in dermatology, may esthetic and cutaneous surgery be learnt as a sub-specialty.

